# Challenges for conducting and teaching handovers as collaborative conversations: an interview study at teaching ICUs

**DOI:** 10.1007/s40037-018-0448-3

**Published:** 2018-09-05

**Authors:** Nico F. Leenstra, Addie Johnson, Oliver C. Jung, Nicole D. Holman, Lieuwe S. Hofstra, Jaap E. Tulleken

**Affiliations:** 1 Department of Critical Care, University Medical Center Groningen, University of Groningen, Groningen, The Netherlands; 2 Department of Psychology, University of Groningen, Groningen, The Netherlands; 3 Department of Anesthesiology, University Medical Center Groningen, University of Groningen, Groningen, The Netherlands; 4 Department of Intensive Care, Martini Hospital Groningen, Groningen, The Netherlands; 5 Department Intensive Care Medicine, Scheper Hospital, Emmen, The Netherlands; 6 Department of Critical Care, University Medical Center Groningen, University of Groningen, Groningen, The Netherlands

**Keywords:** Patient handover, Patient safety, Communication skills

## Abstract

**Introduction:**

Whereas medical shift handovers are increasingly recognized to fulfil important functions beyond information transfer, studies suggest that shift handovers continue to be variably used for reflection, learning or discussion. Little is known of the dynamics of incorporating such functions into ICU shift handovers, resulting in a challenge for the design of educational programs whose underlying philosophies align with the specific requirements of the ICU.

**Methods:**

Intensivists, residents and fellows (*n* = 21) from three ICUs were interviewed to determine perceptions of handover functionality and the boundaries to what must or can be achieved in handover conversations. Interviews were analyzed to isolate training requirements and factors that challenge interactions.

**Results:**

The analysis revealed that ICU physicians value three functions for shift handovers: information transfer, enhancing shared understanding and decision-making, and learning. The functions towards which physicians are oriented were found to be affected by situational characteristics of cases, individuals, teams, and the unit workflow. Whereas some factors are helpful cues for determining communication needs, others raise dilemmas and misaligned expectations with regards to what can be achieved in the handover.

**Discussion:**

Our findings add to the growing case for the education of handovers in complex settings to involve more than information transfers. As residents gain experience, training should be gradually shifted towards more fluid and adaptable approaches to the handover and residents’ ability to engage in joint reflections and discussions. Challenges for engaging in such interactions need to be alleviated, in order to allow the redefinition of handovers as potential sources of safety and learning, rather than error.

## What this paper adds

Whereas it is recognized that shift handovers can fulfil important functions beyond information transfer, studies suggest that they continue to be variably used for reflection, discussion or education. Little is known of the dynamics and challenges of incorporating such functions into ICU shift handovers. We identified ICU physicians’ perceived functionality of shift handovers and the ensuing requirements for handover interactions. Importantly, we identified the factors that challenge their engagement in these interactions. We discussed how our findings can inform the design of a more comprehensive and context-specific system for teaching resident handover skills in the ICU.

## Introduction

Shift handovers are challenged by the potential of communication failures and thereby create a risk to patient safety and quality of care [1–4]. Meanwhile, immediately upon graduation, junior physicians and residents are expected to conduct and participate in ICU shift handovers. Without training in communication skills, they may not be equipped to adequately hand over patients with complex conditions [5]. Whereas formal requirements for handover training have been introduced into medical education [6], a comprehensive and context-specific system for teaching resident handover skills in the ICU is lacking.

A growing number of training courses shift the emphasis in teaching from handovers as one-way information transfers towards collaborative dialogues [7–9]. This approach emphasizes that communication failures can best be ameliorated through loosely structured, two-way handover interactions, allowing for the ‘co-construction’ of understanding [7, 10–16]. It also emphasizes that practitioners need to tell the story of the patient in a manner that fits the singularity of cases and other context factors, such as inexperience [10, 13, 17–19]. Furthermore, there is a growing case for handovers in complex settings being more than information transfers. Training should include teamwork skills that will advance macro-cognitive functions (i. e., the processes by which teams generate new knowledge for addressing unique problems [20]) of handovers, such as re-evaluating situations, reviewing options, and ‘co-orienting’ for future sense- or decision-making [10, 17, 21–25].

However, from reviews of current practice it appears that the incorporation of functions beyond information transfer proves challenging. For instance, a number of studies show that critical assessments are not provided consistently [14, 26]. Whereas some reports [13] demonstrated that a lack of assessment was compensated for by questions from the receivers, others have found that no questions at all were asked in over half of the handovers [14, 15]. Moreover, some studies show that if questions are asked, only few involve critical questions regarding the underlying assumptions of plans [16] or that they often come as subtle questions rather than clear expressions of doubts or alternative perspectives [27].

The importance—and challenge—of targeted training of collaborative interactions may be particularly salient in the ICU: the inherently complex and variable nature of work make it difficult to provide guidelines for the timing and manner with which macro-cognitive functions are to be served. However, little is known of the dynamics of incorporating these functions into the handover, resulting in a challenge for the design of educational programs.

One explanation for the challenge of engaging in collaborative interactions may be inferred from the fact that handovers are traditionally viewed as information transfers, and that their quality is defined by efficiency and reliability [28]. In fact, formal bodies such as the Royal College of Physicians and Surgeons of Canada recently issued requirements which tend towards the teaching of standardized handover schemes to enhance reliability [6]. The predominate focus on the quality of presentations in this approach is in contrast to the more interactional-oriented approaches that target multiple functions of communication [10, 11, 24, 25]. Moreover, there are concerns that with time pressure or high complexity, the handover may be reduced to a paradigm of ‘going down the list’ [29] rather than of ‘making sense of the situation’. It is not unlikely that a similar dichotomy is currently reflected in physicians’ day-to-day negotiation of handover practices.

In this study, we interviewed intensivists, fellows and residents in three teaching ICUs to better understand the timing and manner with which macro-cognitive functions are to be served within ICU shift handovers. We also asked them to describe their challenges for engaging in the associated collaborative interactions. A better understanding would inform the design of educational programs whose underlying philosophies align with the specific requirements of the ICU.

## Methods

### Study setting

Study sites were the ICUs of our university medical centre (UMC; hospital 1, 46 beds in four locations) and two district teaching hospitals (hospital 2 and 3, with 16 and 13 beds respectively), all in the Netherlands. At all sites, six to ten patients are handed over three times a day at designated times with protected time: 30 minutes at hospital 1 and 3, and 60 minutes at hospital 2. Handovers are given predominantly by supervised residents. All scheduled staff of the incoming and outgoing teams are present, varying from between 3 and 11 physicians in total, of which approximately half are trainees. At the two general district hospitals, handovers take place in a designated quiet room. At the UMC, locations are designated rooms or patients’ bedsides.

### Interviews

Semi-structured interviews with individual participants (12 intensivists, 3 fellows and 6 residents) were conducted between October 2013 and April 2014. The interview protocol was targeted at identifying the timing and manner with which macro-cognitive functions are to be served within ICU shift handovers, and the factors that affect or impede their engagement in these interactions. Participants were first asked to describe what they perceive to be the functions of the handover, in order to identify individuals’ underlying values for the handover process. Then they were asked to describe the circumstances in which the importance of each function might be triggered. Next, they reflected on whether to adapt their presentations to accommodate certain circumstances. In order to explore the perceived requirements for two-way interactions, we then asked them to describe the behaviours of both receivers and senders they find of use to the functions mentioned earlier in the interview. In this section, we also asked for factors that challenge teamwork interactions and for their conceptions of the boundaries to what must and can be achieved in the handover. During the course of the interviews, probes were added based on preliminary analysis of initial interviews. Interviews lasted 40–60 minutes and were transcribed verbatim.

### Interview analysis

A qualitative ‘conventional’ content analysis was performed on the data following the procedural steps as summarized by Hsieh and Shannon [30]. We felt that the divergence in the literature on handover education and the limited understanding of the complexities of ICU shift handovers justified an inductive approach.

Interview transcripts were de-identified prior to the analysis. Transcripts were first read and segments of interest were then coded using keywords derived from the segments, using ATLAS.ti (Scientific Software Development, Germany). These codes would function as short summaries of the segments of interest. Codes were generated and applied as appropriate across interviews and continued to be generated as necessary across the coding session. These steps were repeated after every three to four interviews to allow insights to develop and have probes added for subsequent interviews. This procedural element from the grounded theory approach [31] was added, given the method’s iterative approach to gathering insights and model building. In order to make sure that the coding process was performed reliably, inter-rater reliability was determined by calculating the percentage of consistently coded segments of the first rater with a second rater for the first three interviews (80.4% of 347 segments). After agreement was reached on how to complete coding, the remaining interviews were coded by the first author. The codes from all interviews were then sorted into categories (e. g., receiver behaviours; team factors) and grouped into meaningful clusters (i. e., by handover function). The codes and the original interview segments were reviewed to identify key messages, nuances and contradictory perspectives. Summaries of the analysis were reviewed by a panel of the interviewed respondents for internal validation.

### Sampling

At the UMC, participants volunteered on the basis of email invitation. At the two district hospitals, the heads of department introduced the study to their staff and provided us with an interview schedule with volunteers. At least two intensivists and two residents were sampled from each study site to maximize the diversity of perceptions. Participants were added until interviews failed to reveal new insights. Participants gave written informed consent. Ethics approval was waived by the institutional review boards of the UMC Groningen and the district hospitals.

## Results

The analysis of perceived functionality revealed that ICU shift handovers were considered essential in order to make sure important aspects of the case are highlighted and well understood by the receiver, and to coordinate care activities across shifts. Additional functions included enhancing shared understanding and decision-making, and individual and organizational learning. The functions towards which physicians are oriented were found to be affected by characteristics of cases, individuals, teams/organizations, and the workflow at the unit (Tab. 1). Whereas some of these factors (e. g., diagnostic uncertainty) serve as helpful cues to collaboratively set aims, other factors present physicians with dilemmas and misaligned expectations with regards to what must and can be achieved. In the following section, the conditions under which each of the identified functions is encouraged are identified, as are the challenges faced in engaging in the associated behaviours.

**Table 1:** Factors that moderate or challenge collaborative communication needs during ICU shift-handovers

Case characteristics	– Diagnostic uncertainty – Complexity – Treatment progress
Individual characteristics	– Experience – Familiarity with patient – Handover preferences (own and others’) – Treatment preferences (own and others’) – Orientation towards personal agenda (e. g., appearing competent or selling a point of view) – Weariness
Group characteristics	– Group size – Distribution of experience – Familiarity with patient – Organizational values/culture regarding handover utility – Social safety (e. g., ability to ask questions)
Workflow characteristics	– Formally available time – Time of handover – Number of patients to hand over – Urgency of patient care and overall workload – Opportunities to discuss patients at other times during the shift

### Function #1: Transferring awareness and understanding of the patient’s problems and care plans

Whereas the function of transferring understanding of the case and the plan from one team to the next and the coordination of tasks across shifts is the same for every handover, situational differences affect physicians’ perception of what is required to transfer understanding successfully. One such factor is case complexity and case uncertainty. Complex and uncertain cases require a more comprehensive presentation including arguments and explanations so that colleagues can reflect and fully understand the problem. Also, more elaboration or details may be given if they are functional to illustrating the gravity of the situation.

However, respondents did note that finding the appropriate balance with regards to the amount of elaboration can be challenging (see Tab. 2 for examples of interview segments). Too little detail may leave open questions about the diagnostic theories and how they led to the treatment plan, whereas too much may obscure the main message and leave insufficient time available for other patients.

**Table 2:** Examples of interview segments that illustrate the choices and challenges associated with the function of transferring understanding and awareness of the patient’s problems and care plan

Challenges	Examples of interview segments
An appropriate amount of detail to illustrate or support the story must be communicated	‘Your assessment of the case, especially the difficult ones, should be supported with arguments and findings so that your colleagues can reflect and fully understand the problem.’ Intensivist, hospital 3
	‘I think it’s challenging, being concise to keep everybody focused but at the same time adding enough detail so people will understand why things happened the way they did.’ Intensivist, hospital 1
	‘In many situations, you don’t need the details such as respiration rate and support settings if you provide the right context and a good summary. […] For instance, if a patient who has had heart surgery […] keeps bleeding, and a lot is required to keep pressures up, you’ll be illustrating the gravity of the situation with some numbers.’ Intensivist, hospital 1
	‘I know generally what kind of handover to expect from my colleagues. A concise handover from one; from another a handover with details that I personally do not think are relevant, or with considerations I would not mention […] One person has lots of confidence, the other has less, and that seeps through in their handovers.’ Intensivist, hospital 1
	‘What I find important may not be what my colleague finds important. So I recommend [residents] to do one thing. The next day my colleague instructs them otherwise. For [residents] it is very hard knowing when you are doing it right. And that is the point, you never can.’ Intensivist 4, hospital 1
	‘We hand over 20 to 24 patients […]. That means I cannot memorize 10 details per patient, I only have a limited number of mental slots per patient available.’ Intensivist 6, hospital 1
Handovers must be adapted to receiver experience to an appropriate extent	‘The receiver determines how you hand over. […] When I hand over to a staff member, […] I outline the patient in more abstract terms. When the receiver is less experienced, I provide the same outline, only with more explanations of the how’s and why’s, and the pitfalls along the way. For instance, I could point out that ventilation problems of a patient with COPD are not resolved simply by increasing ventilation pressures, as you normally would. I do this to prevent the development of unnecessary problems or harmful events.’ Fellow, hospital 1
	‘As a resident it’s nice to know the daily ins and outs, as I will need to handle those things. A supervisor is more interested in the overall goals and long-term plans.’ Resident, hospital 1

Whether different expectations between individuals result in difficulties for establishing effective interactions varied. In some hospitals (particularly at the smaller hospital 3), respondents said that they did try to anticipate the receivers’ preferred level of detail, adjusting their presentation to whether the receiver tends to be more concerned with the details of plan execution or to be more reflective. Others (particularly residents in hospital 1) reported being confronted with frustration and impatience if they were perceived as being too detailed. These differing preferences among colleagues were reported to affect residents’ learning: residents receive varying and contradicting feedback regarding preferred handover behaviours and content across supervisors.

Another important factor that affects how physicians communicate information is the experience of receivers: the level of abstraction in the explanation and the specification of actions should be tailored to the receivers’ knowledge base and experience. A challenge for larger teams (as at our hospitals 1 and 2) is that it is not always possible to fully disclose all the details residents may need or to fully include residents in discussions, because of time constraints. For instance, if, in complex cases, discussions are needed, the educational aims of the handover are set aside. Residents are aware of these constraints: they would look things up or save questions for after the handover in case of time pressures or if they feel the atmosphere is not conducive to questions. Furthermore, residents consider whether their questions will benefit the rest of the audience.

### Function #2: Enhancing shared understanding and decision-making

Respondents felt that joint reflections and discussions could benefit the patient, particularly in rare, uncertain or complex cases. Associated receiver behaviours included cross-checking, asking for senders’ reasoning, and introducing alternative perspectives. Associated sender behaviours included communicating alternate considerations, doubts or hunches and reflecting on information that is indicative and counter-indicative of diagnoses.

Although such reflection-initiating behaviours can potentially help all members to understand the situation, help catch errors and decide how to proceed, respondents also noted the drawbacks of initiating discussions (Tab. 3). Drawbacks included that discussions might drift away from the main problems and that less time is left available for other patients. The relevance of the discussion to patient outcome was considered an important indicator of the appropriateness of the behaviour. It was also argued that discussions in the handover do not necessarily add new perspectives to those that were generated during consults in the previous shift. Respondents were aware that colleagues held differing views on the functionality of reflection in the handover, and noted that getting the other to join in collaborative reflections would sometimes require some determination. With regards to decision-making, it was often suggested that, given that problems are often too complex to solve in the handover, it is sufficient to inventory colleagues’ perspectives and save actual decision-making for the multidisciplinary round.

**Table 3:** Examples of interview segments that illustrate the choices and challenges associated with the function of enhancing shared understanding and decision making

Challenges	Examples of interview segments
Doubts, suspicions and considerations must be communicated to an appropriate extent	‘[Omitting suspicions] can be dangerous: If there’s a suspicion of internal bleeding in the stomach but this is not communicated across the shift, and your colleague finds a thrombotic leg and starts anticoagulants, the patient might die.’ Intensivist 1, hospital 2
	‘If you don’t communicate your uncertainties, where you’re at in making sense of the situation, chances are that your colleague will spend another 4 to 6 hours before reaching the same level of understanding that you had. So tell them, ‘I don’t know. I’ve considered this and this, it’s not improving’.’ Fellow 2, hospital 1
	‘I do report if I’m not sure of something. I’d nuance the strength of my diagnosis. […] But I wouldn’t express too many of my suspicions as I might bias someone towards a certain direction, which may not be wise since it’s only my perspective.’ Fellow 1, hospital 1
	‘It’s especially dangerous when handovers consist of overly ‘polished’ reports and justifications; to appear more competent, to avoid discussions, or to ‘sell’ a point-of-view. ’ Intensivist 3, hospital 1
	‘An experienced sender knows exactly how to control a handover, what to tell and what not to in order to start or avoid a discussion. […] If one presents himself being so certain, it’s difficult for the audience to wonder ‘what if it’s not …?’ […] The real art of handing over is to have a vision, but not to push your point too heavily.’ Fellow 2, hospital 1
There must be ample opportunity to reflect and debate	‘I think it’s good to discuss certain patients with the new team: ‘couldn’t this be the problem, or this, or should we try this, or this?’ I know not everybody’s a big fan of this.’ Intensivist 2, hospital 2
	‘Since we frequently do our shifts alone, the handover offers a good opportunity to test your plans or diagnoses with a colleague, and you can discuss your decisions for the complex cases.’ Intensivist 2, hospital 3
	‘Discussions are about the things of importance: about starting or stopping an intervention, ‘have you considered this or this?’. It’s not about prescribing aspirin, it should be about the things that could make a difference. ’ Intensivist 2, hospital 2
	‘It is often the staff members who initiate a dialogue: ‘why have we done this, should we try this as well?’ […] But it leaves me thinking: ‘we’ve gone through this already during the multidisciplinary rounds’. […] A fresh perspective could be good, but often it’s too much, nothing new comes up and discussions drift away from what is immediately important.’ Resident 1, hospital 2
	‘I still find it difficult to balance the desire to address things that could have been done differently, and the desire to have someone going home with the feeling they have […] done a good day of work.’ Intensivist 3, hospital 1
	‘I have had times [as a resident] in which I felt others were accusing me of poor judgment, for instance, when they asked me why I chose option A rather than B. I immediately assumed they felt I should have gone for option B. […] I think the problem is for the most part the recipient of the question, who is tired and feels vulnerable after a long shift.’ Intensivist 4, hospital 1

Similarly, respondents were not unanimous with regards to the utility of voicing suspicions. Whereas some argued that omitting suspicions could result in dangerous situations when complications are not anticipated, others reflected that communicating suspicions may bias an otherwise fresh perspective. On the other hand, some respondents reported the—limited—occurrence of ‘overly polished’ reports, in which senders provide a self-consistent, tidy report of what was done and why it was justified. Such reports would benefit senders’ personal agendas of appearing more competent, avoiding discussions or of ‘selling a point of view’, but would also cut short discussion that might otherwise contribute to a more positive patient outcome.

Another challenge to engage in joint reflections was a perceived difficulty to ask critical questions while maintaining a positive atmosphere. Questions can very easily be misinterpreted for their intent, as was illustrated in examples where senders felt they were being accused of poor judgment, rather than being invited to enhance shared understanding.

### Function #3: Individual and organizational learning

Few respondents reported that teaching (e. g., asking questions to stimulate residents’ knowledge synthesis) was a formal function of the handover (Tab. 4). Instances were also reported in which handovers were used to learn as a unit or to strengthen shared values, for instance, when something had almost gone wrong. Both forms of learning were reported to occur only if the number of patients, the available time and the number of attendants for whom the teaching would be relevant allowed it. As a consequence, residents’ learning was described as a by-product of the handover occurring, for instance, by listening to staff’s discussions. Moreover, the educational aims of the handover are set aside if, for instance, important discussions are needed. Teaching was more likely to occur during handovers in the evening, where fewer staff members were present and the workloads for the upcoming shift were likely to be lower. In hospital 3, it was also said that the small group size (3–4 physicians) provided a low threshold for teaching to occur.

**Table 4:** Examples of interview segments that illustrate the choices and challenges associated with the function of individual and organizational learning

Challenges	Example interview segments
There must be ample opportunity to incorporate teaching	‘If the case is so complex that discussions are needed, the educational aims of the handover are set aside. If the patient will benefit from the discussions being held, this becomes more important than ensuring the lesser experienced resident entirely gets it.’ Fellow, hospital 1
	Sure, you must never be afraid [to ask questions]. That is very important as a young resident, when everything is new. You might doubt, ‘is it okay to do so?’, but asking questions is never wrong. Resident 1, hospital 2
	‘Sure sometimes things are not always clear to me, like ‘why is this person given this type of antibiotics and not this one?’ Or ‘why was this chosen and not that?’ But I will not always ask. Often, you can look things up. It depends on the atmosphere and the time pressure.’ Resident 3, hospital 2
	‘We are limited: depending on how many patients we have, we need all the time we have to go by all patients. But if there is time, and there is a problem where more than one person can learn from […] than the handover might be the best time to offer some teaching. Provided that you stay within the time slot.’ Intensivist 7, hospital 1
There must be ample opportunity to incorporate organizational learning and strengthening of shared values	‘I wanted others to learn from my experience and said ‘that is why we want such patients going straight to the OR and not to us first.’ […] I wanted to make sure we all agree that we cannot let things like this happen again. But, although I think that we as a team should discuss such things, you could also question whether the handover is the right time. It can be distracting, and if you’re not careful, it will take up a lot of time.’ Intensivist 3, hospital 1
	‘Because I think communication should always lead to doing the right thing. That means we must incorporate learning into our communication. If we do this structurally, we will reach a higher level as a team, and patients will profit from this. If you use communication only to manage care […] you will only be putting out fires.’ Fellow 1, hospital 1

## Discussion

In order to inform the design of educational programs for residents’ ICU shift handovers, we sought to further the understanding of the timing and manner with which macro-cognitive functions need to be served within shift handovers. We discuss the requirements for the conduct and teaching of handovers in the ICU first, followed by a discussion on the current challenges for engaging in collaborative interactions.

### Requirements for handover training in the ICU

We found that physicians adapt their presentations in order to accommodate complexity, ambiguity or uncertainty in cases: physicians discuss in more detail their assessment of the patient’s problems and how problems are interrelated; their interpretation of diagnostics; and of how treatment success might relate to the diagnoses. Such presentations include more explanations, reflections and details than those of simpler cases. This aligns with previous studies in which assessments of complex and ambiguous cases consistently differed from those of simpler cases [13, 18]. Our findings resonate with the suggestion that handing over patients is more than presenting ‘facts and figures’ [13] and rather requires flexible ways of telling the story [10, 17–19, 32], skilful verbalization of one’s clinical reasoning and careful expression of uncertainties and options [13, 18] and the provision of details ‘on demand’ [25, 29].

Our finding that variations in handover presentations are a necessity could be seen to raise questions about the current gravitation towards standardized communication structures. A matter of concern is that too rigid communication schemes may fail to appreciate the need for such variation [17, 24]. This notion was confirmed in a recent evaluation study of the I‑PASS mnemonic educational program: residents experienced a challenge between the program’s focus on mnemonic adherence and their ability to use their own judgment in differing circumstances [33]. On the other hand, junior residents can fall back on standardized communication schemes if they lack a more holistic understanding of a patient’s case [8, 17, 29]. Our findings could therefore be seen to imply that teaching a basic structure is a starting point rather than the endpoint. Supplemental training should address flexible narrative approaches that help to synthesize clinical information, express clinical reasoning, and carefully give details at points in the story where their relevance becomes clear.

In addition to transferring understanding, our respondents valued shift handovers as opportunities for jointly reflecting on the patient’s situation and for jointly orienting on future decision-making and planning. These findings are consistent with previous notions that shift handovers in complex settings are an important means to enhance the resilience of patient care [5, 13, 25, 27, 34–36]. For instance, a recent study showed that cross-covering ICU fellows were more vigilant and made more decisions when compared with continuity-of-care fellows, and thereby reduced mortality [37]. The authors suggest that “the balance between medical errors from handovers and the benefit of a ‘second look’ seems to favour the latter” [37].

Handover training in the ICU should thus advance important non-technical skills. Currently, handover programs are often limited to receivers’ behaviours that help enhance reliability, such as read-backs [8, 12, 38, 39]. Fewer programs target skills for enhancing handovers as a ‘resiliency activity’. Our findings add to the growing case for handover training also addressing skills of exploring the previous team’s sense-making, introducing concerns, suggesting alternative options and discussing anticipated problems.

### Understanding the challenges of engaging in collaborative conversations

We found that certain situational factors, such as high case complexity, serve as helpful cues for physicians to determine communication needs, as has been suggested in previous work [18]. However, we found that the simultaneous influence of other factors creates dilemmas for physicians with regards to what must and can be achieved in the handover. Often, dilemmas involved trade-offs between efficiency and thoroughness due to time pressure or a high number of patients to hand over. This trade-off was often reflected in physicians’ tendency to prioritize the function of information transfer over joint reflections or discussions and, particularly, education. However, the pressure to be concise and clear-cut may even hamper the goal of allowing all members to fully achieve understanding of the case.

That colleagues hold misaligned expectations might be explained by the theory that physicians make their own trade-offs depending on how they perceive the factors within the context and how they value the benefits and drawbacks of behaviours within the situation [40]. One way of better coordinating handover dialogues may then be for teams to communicate more explicitly about the desired outcome of the conversation. Team training could be targeted at such interpersonal coordination skills. Also, team training could facilitate consensus finding regarding the boundaries for handover dialogues. A number of issues seem to be of particular interest: systems for identifying which patients might benefit from more extensive discussions; systems for allocating time across patients; and how joint reflections and discussions during handovers add to previous consultations and rounds.

The flexible and variable conceptions of intensivists may leave residents uncertain with regards to best practices. Residents observe a wide range of handover styles and receive feedback that may be contradictory. Without reference to consideration of, for example, the characteristics of the situation or the benefits and drawbacks of certain behaviours, residents will not fully appreciate the values that underlie a behaviour or how the selection of information may or may not apply to other handovers [41].

Our finding that learning was more often seen as a by-product is in stark contrast to views that handovers are ideal platforms for case-based learning [42, 43]. On the other hand, our respondents’ hesitation about engaging in teaching activities included careful consideration of patients’ needs and practical constraints of time. This hesitation closely resembles findings from a survey among intensivists at academic teaching ICUs that increased clinical workloads are associated with insufficient time for patient care and teaching [44]. More efficient ways of incorporating education into daily care activities are needed [45]. Shift handovers may offer one such opportunity because they involve cycles of action, reflection, planning and adaptation within care trajectories that closely resemble learning cycles such as Kolb’s [42].

## Conclusion

Our findings add to the growing case for the education of handovers in complex settings to involve more than information transfers. Teaching a standardized communication scheme can be a helpful starting point but, as residents gain experience, training should be gradually shifted towards more fluid and adaptable approaches to the handover. Further, training should target residents’ ability to engage in collaborative conversations that help advance resilience in high-stakes care. Challenges for engaging in such interactions need to be alleviated in order to allow the redefinition of handovers as potential sources of safety and learning, rather than of error.
